# Clinical Characteristics of Parkinsonism in HTLV‐1‐Associated Myelopathy

**DOI:** 10.1002/acn3.70121

**Published:** 2025-06-27

**Authors:** Mika Dozono, Satoshi Nozuma, Shota Hirakata, Takashi Yoshida, Daisuke Kodama, Masakazu Tanaka, Eiji Matsuura, Ryuji Kubota, Hiroshi Takashima

**Affiliations:** ^1^ Department of Neurology and Geriatrics Kagoshima University Graduate School of Medical and Dental Sciences Kagoshima Japan; ^2^ Division of Neuroimmunology, Joint Research Center for Human Retrovirus Infection Kagoshima University Kagoshima Japan

**Keywords:** HAM/TSP, HTLV‐1, parkinsonism

## Abstract

**Objective:**

Human T‐lymphotropic virus type 1 (HTLV‐1)‐associated myelopathy/tropical spastic paraparesis (HAM/TSP) is the classic neurological manifestation of HTLV‐1 infection; however, this virus has also been associated with other neurological disorders. Concurrent parkinsonism is relatively rare and presents diagnostic challenges. The present study aimed to identify the clinical characteristics of HAM/TSP with parkinsonism.

**Methods:**

This retrospective study included HAM/TSP patients hospitalized in Kagoshima University Hospital from January 2000 to March 2022. Clinical and laboratory findings of the HAM/TSP patients with parkinsonism (P‐HAM) were collected from the medical records and compared with HAM/TSP patients without parkinsonism (typical HAM/TSP [T‐HAM]). P‐HAM cases were defined as patients presenting with any combination of rigidity, resting tremor, bradykinesia, and/or postural instability, with these symptoms not attributed to HAM/TSP.

**Results:**

Of 246 HAM/TSP patients, 11 (4.5%) presented with parkinsonism. Compared with T‐HAM, the age of onset was significantly older (65.0 vs. 48.8 years, *p* = 0.001) in patients with P‐HAM. Moreover, despite a shorter illness duration (8.5 vs. 12.5 years, *p* = 0.151), the Osame Motor Disability Score was significantly higher in P‐HAM cases than in T‐HAM cases (6.3 vs. 4.6, *p* = 0.0132), and all P‐HAM cases had scores ≥ 4. Laboratory findings showed no differences between the groups.

**Interpretation:**

In our cohort, 4.5% of HAM/TSP patients had concomitant parkinsonism, which was associated with a later age of onset and greater disease severity. The coexistence of parkinsonism in HAM/TSP may be underrecognized, and our findings expand the clinical spectrum of neurological disease with HTLV‐1 infection.

## 1 Introduction

Human T‐lymphotropic virus type 1 (HTLV‐1) was the first human retrovirus to be identified and infects an estimated 5 to 10 million people worldwide [1]. In Japan, it has been estimated that there were approximately 535,000 carriers [2]. The majority of infected individuals remain asymptomatic carriers throughout their lifetime; however, some develop HTLV‐1‐associated myelopathy/tropical spastic paraparesis (HAM/TSP), adult T‐cell leukemia/lymphoma (ATL), or inflammatory conditions such as uveitis or dermatitis [3]. HAM/TSP is a progressive neurological disorder that is characterized by spastic paraplegia, urinary disturbance, and sensory impairment [4, 5]. Regions with high endemicity for HTLV‐1 are restricted to the Caribbean, South America, Africa, the Middle East, Australia, Melanesia, and Japan [6]. However, recent rapid globalization has led to a redistribution of HTLV‐1 infection driven by immigration and migration from endemic regions to urban areas [7]. Consequently, opportunities to diagnose and treat HTLV‐1‐associated diseases may arise even in non‐endemic regions.

Parkinsonism is a clinical syndrome that is characterized by a tetrad of resting tremor, rigidity, bradykinesia, and postural instability. The most common form of parkinsonism is Parkinson's disease (PD), a progressive neurodegenerative disorder that is pathologically defined by the loss of dopaminergic neurons in the substantia nigra as well as the presence of protein inclusions (termed Lewy bodies). It has been estimated that there were 6.1 million Parkinson's disease patients worldwide and 256,000 in Japan [8]. Although numerous genes have been identified as causes of or risk factors for PD development [9, 10], most cases are sporadic. This suggests the involvement of complex interactions between genetic predispositions and risk factors such as aging, lifestyle, and environmental exposures. Moreover, recent studies have shed light onto how inflammation and immune dysfunction may play a role in PD development and prognosis [11].

The hypothesis of an infectious etiology in PD emerged from observations of the incidence of post‐encephalitic parkinsonism following the 1918 influenza pandemic [12]. Since then, epidemiological and basic studies have suggested a potential association between bacterial or viral infection and parkinsonism [13, 14, 15, 16]. Notably, certain infectious pathogens can be neurotropic and directly damage the nigrostriatal pathway, or can indirectly synergize with the immune system to trigger neuroinflammation, leading to parkinsonism.

Although the classic neurological manifestation of HTLV‐1 infection is HAM/TSP, other neurological disorders, including myopathy, polyneuropathy, cerebellar ataxia, and cognitive deficits, have been associated with the virus [17, 18, 19]. Our institute manages many HAM/TSP patients; however, cases with concurrent parkinsonism are rare and present diagnostic challenges. Furthermore, because only two case reports of HAM/TSP patients with parkinsonism have been published [20, 21], the association between HAM/TSP and parkinsonism and its associated characteristics remains unclear. People infected with HTLV‐1 have increased mortality, and the spectrum of diseases attributable to the virus may be more extensive than previously recognized [22, 23].

The present study aimed to identify the clinical characteristics of HAM/TSP patients with parkinsonism (P‐HAM). To do this, the clinical and laboratory findings of patients with P‐HAM were compared with those of patients with HAM/TSP without parkinsonism (typical HAM/TSP [T‐HAM]).

## 2 Methods

### 2.1 Ethics

This retrospective study was approved by the Institutional Review Board of Kagoshima University (IRB protocol no. G491). All participants provided written informed consent.

### 2.2 Subjects

This retrospective study included HAM/TSP patients admitted to Kagoshima University Hospital from January 2000 to March 2022. All patients with HAM/TSP were diagnosed according to the World Health Organization diagnostic criteria [24]. We collected clinical (age, sex, age of onset, initial symptoms, disease duration, and neurological disabilities) and laboratory (HTLV‐1 proviral load [PVL] and CSF cell number, protein, and neopterin) data from the medical records. All data were collected at the time of admission and prior to the treatment. Neurological disabilities were assessed using the Osame Motor Disability Score (OMDS) [25] and Medical Research Council (MRC) sum score. As reported previously [25], the OMDS ranges from 0 to 13, with 3 and 4 scored as follows: 3, cannot run; 4, needs handrails to ascend and descend stairs or the occasional use of a cane. We used a different assessment for the subgroup with OMDS > 4 because these patients require aids in daily living. The MRC sum score is an assessment of limb muscle strength. Given that patients with HAM/TSP do not typically exhibit upper extremity weakness, lower extremity strength was assessed independently using the MRC sum score. A subgroup of patients with rapid disease progression was defined by the deterioration of motor disability by more than three grades within 2 years.

P‐HAM cases were defined as patients presenting with any combination of rigidity, resting tremor, bradykinesia, and/or postural instability, with these symptoms not attributable to HAM/TSP. PD, dementia with Lewy bodies, multiple system atrophy (MSA), and progressive supranuclear palsy were diagnosed according to their respective diagnostic criteria [26, 27, 28, 29]. The final diagnosis was determined by three or more neurologists and was based on the clinical course, neurological findings, and imaging studies of each patient. Control cases were defined as T‐HAM.

### 2.3 Statistical Analysis

Data were analyzed using GraphPad Prism version 10.2.2 (GraphPad Software, Boston, MA). Statistical analyses were performed using a non‐parametric test (the Mann–Whitney test) for continuous variables and the *χ* ^2^ test for categorical variables. Survival was estimated using the Kaplan–Meier method. The final endpoint was defined by an OMDS of 4. Differences were considered significant when *p* < 0.05.

## 3 Results

### 3.1 Clinical Characteristics of Patients With P‐HAM

Of the 246 HAM/TSP patients who were admitted to our hospital between January 2000 and March 2022, 11 patients (4.5%) presented with parkinsonism (P‐HAM). All 11 cases presented with spastic paraplegia and met diagnostic criteria for HAM. Of the 11 patients with P‐HAM, five were diagnosed with PD, two with MSA, two with progressive supranuclear palsy, and one with dementia with Lewy bodies; the remaining patient was unclassifiable. Although some cases were not classified as P‐HAM at admission, neurologists later diagnosed them based on nuclear imaging, treatment responsiveness, and clinical manifestations. Ten patients were treated with levodopa, of whom nine demonstrated improvements in motor symptoms associated with parkinsonism. The following is a detailed description of the two cases we were able to follow after discharge.

#### 3.1.1 Patient 1

**FIGURE 1:**
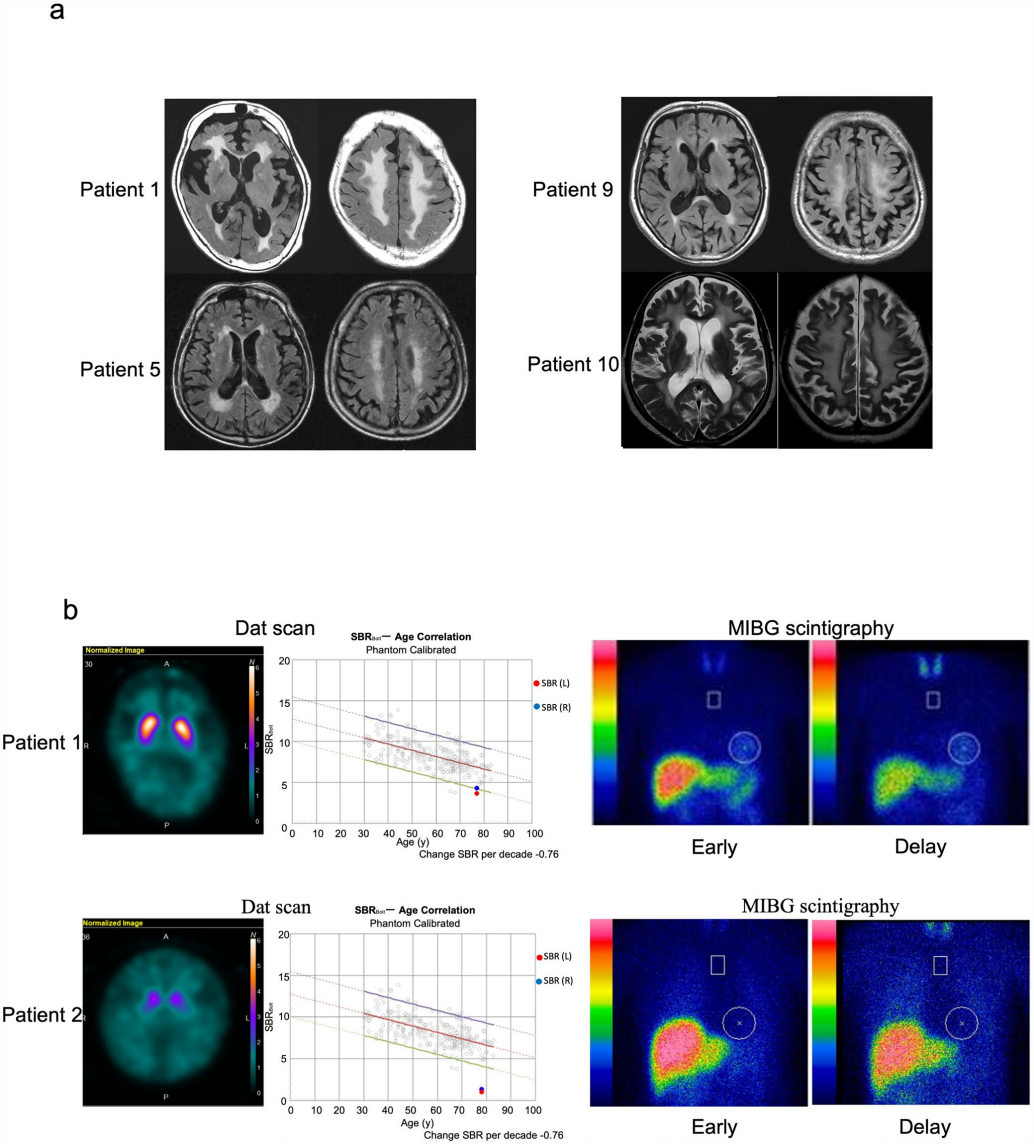
MRI, DAT scans, and MIBG scintigraphy in HAM/TSP patients with parkinsonism. (a) Brain MRI from four patients. White matter abnormalities in the cerebrum were frequently observed. (b) DAT scans and MIBG scintigraphy. The DAT scans in Patients 1 and 2 indicated decreased bilateral DAT uptake. MIBG scintigraphy in Patient 1 showed normal uptake. In Patient 2, both the early and delayed MIBG H/M ratios were significantly low. DAT, dopamine transporter; HAM/TSP, human T‐lymphotropic virus type 1‐associated myelopathy/tropical spastic paraparesis; H/M, heart‐to‐mediastinum; MIBG, metaiodobenzylguanidine.

Patient 1 was a 77‐year‐old woman who became aware of gait disturbance at the age of 76 years. She fell down a lot and began using a cane a few months later. One year later, she was admitted to our hospital. On admission, proximal lower extremity muscle weakness, spasticity of the lower extremities, brisk reflexes, and positive Babinski's sign were observed. She was also noted to have rigidity and postural dysreflexia and walked with small steps. Anti‐HTLV‐1 antibodies were detected in both the serum and CSF. Brain MRI demonstrated cerebral white matter lesions (Figure 1a). A dopamine transporter (DAT) scan revealed decreased uptake in the bilateral putamen (specific binding ratio [SBR]: right, 4.30; left, 3.68 [lower limit of the 95% prediction interval of SBR in the 70–79 age group: 4.42]). Metaiodobenzylguanidine (MIBG) scintigraphy revealed a normal uptake and washout rate (heart‐to‐mediastinum [H/M] ratios: early, 2.90; delayed, 2.83 [lower limit of our institute: 2.2]; washout rate, 15.3%) (Figure 1b). The patient was diagnosed with a complex case of HAM/TSP and MSA. She received IV methylprednisolone (1000 mg/day for 3 days) to treat HAM/TSP, resulting in improvements in lower extremity muscle strength, gait stability, and walking speed. In the 10‐m walk test, her time improved from 28 s (pre‐treatment) to 13 s (post‐treatment). The patient also received levodopa treatment for her parkinsonism; however, there was no noticeable improvement. Moreover, despite no changes in lower extremity muscle strength after discharge, her gait disturbance and postural instability rapidly worsened.

#### 3.1.2 Patient 2

Patient 2 was an 81‐year‐old woman with a history of depression. She had experienced bradykinesia, tremor, and gait disturbance from the age of 69 years. She was diagnosed with PD at the age of 72 years; 5 years later, she had spasticity of the lower extremities, narrow steps, and postural instability. Although her medications to treat PD were adjusted, her symptoms did not improve. At the age of 79 years, she was referred to our hospital for admission. Her neurological examination revealed a masked face, rigidity of the upper extremities, spastic paraplegia, tremor, urinary disturbance, gait freezing, and postural instability. Anti‐HTLV‐1 antibodies were detected in both the serum and CSF. Brain MRI demonstrated some age‐appropriate white matter lesions (data not shown). A DAT scan revealed decreased uptake in the bilateral putamen (SBR: right, 1.37; left, 1.06 [lower limit of the 95% prediction interval of SBR in the 70–79 age group: 4.42]). MIBG scintigraphy revealed a decreased uptake and a high washout rate (H/M ratio: early, 1.60; delayed, 1.25 [lower limit of our institute 2.2]; washout rate, 63.2%) (Figure 1b). The patient was diagnosed with a complex case of HAM/TSP and PD. She received IV methylprednisolone (1000 mg/day for 3 days) to treat HAM/TSP, which resulted in improvements in lower extremity strength and gait speed. After adjusting her medications for PD, her motor disability improved slightly. However, her response to levodopa gradually decreased after discharge, and she experienced frequent falls. Although her muscle weakness did not progress, her gait disturbance worsened. She has been hospitalized for a long period since the age of 81 years.

### 3.2 Imaging Characteristics of Patients With P‐HAM

The clinical and imaging characteristics of the 11 patients with P‐HAM are summarized in Table 1. Rigidity, bradykinesia, and postural instability were observed in all 11 patients, and resting tremor was present in eight patients (73%). Brain MRI findings were available in 10 patients. White matter lesions were observed in nine patients, of whom five exhibited extensive lesions. The brain MRI findings of Patients 1, 5, 9, and 10 are presented in Figure 1a. A DAT scan was performed in three patients, and the SBR was reduced in all cases.

**TABLE 1:** Clinical and imaging characteristics of HAM/TSP patients with parkinsonism.

Patient number	Duration of parkinsonism	Rigidity	Resting tremor	Bradykinesia	Postural instability	Gait freezing or wiggle walk	Hoehn & Yahr	Response to L‐dopa	Brain MRI		MIBG H/M ratio	DaT scan SBR	Definition
									Atrophy	White matter lesion			
1	1	+	−	+	+	+	3	Poor	Basal ganglia, Cerebellum	+	Normal	Low	MSA
2	10	+	+	+	+	+	3	Good	−	+	Low	Low	PD
3	4	+	−	+	+	+	3	Good	−	+	Low	N.A.	PD
4	2	+	+	+	+	+	4	Good	Midbrain	−	N.A.	Low	PSP
5	4	+	+	+	+	+	5	Good	Frontotemporal lobe	+	Low	N.A.	DLB
6	4	+	−	+	+	+	3	Good	−	+	Normal	N.A.	PSP
7	1	+	+	+	+	+	3	Good	−	+	Low	N.A.	PD
8	1	+	+	+	+	+	3	Good	N.A.	N.A.	N.A.	N.A.	PD
9	2	+	+	+	+	+	5	Good	Brainstem, Cerebellum	+	Normal	N.A.	MSA
10	2	+	+	+	+	+	5	Good	−	+	N.A.	N.A.	PD
11	0	+	+	+	+	+	5	N.A.	−	+	N.A.	N.A.	Unclassifiable

Abbreviations: DAT, dopamine transporter; DLB, dementia with Lewy bodies; HAM/TSP, human T‐lymphotropic virus type 1‐associated myelopathy/tropical spastic paraparesis; H/M ratio, heart‐to‐mediastinum ratio; MIBG, metaiodobenzylguanidine scintigraphy; MSA, multiple system atrophy; N.A., not available; PD, Parkinson's disease; PSP, progressive supranuclear palsy; SBR, specific binding ratio.

### 3.3 Comparison of Clinical Features Between P‐HAM and T‐HAM

**FIGURE 2:**
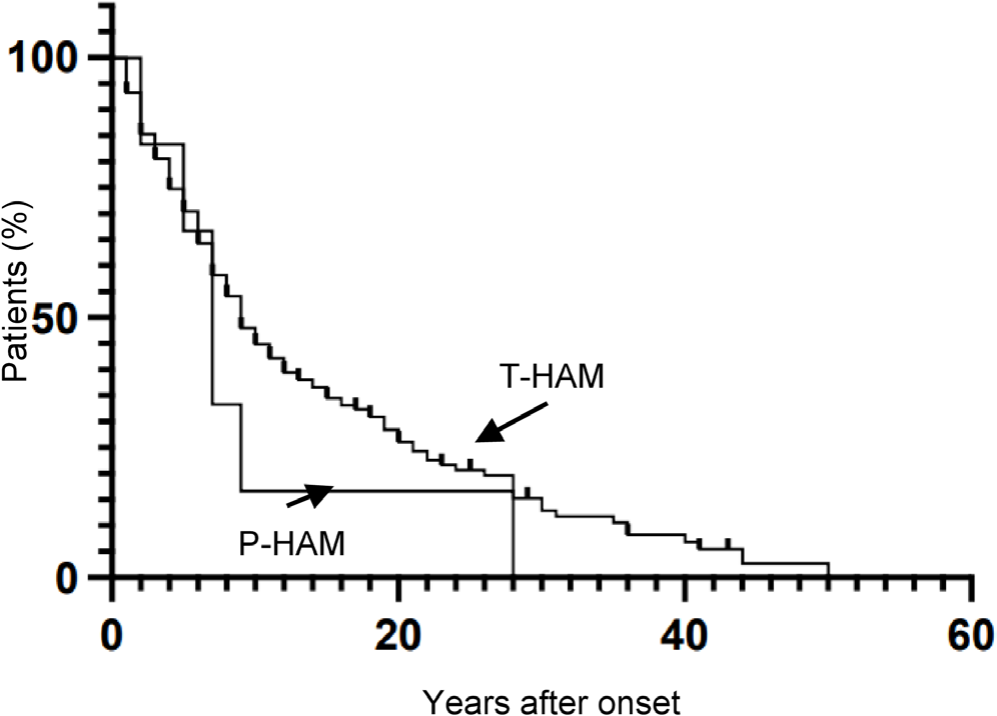
Kaplan–Meier estimates of the time from disease onset to a score of 4 in the OMDS. The time from disease onset to a score of 4 in the OMDS tended to be earlier in patients with P‐HAM (5.3 ± 8.3 years) than in those with T‐HAM (8.9 ± 9.9 years, *N* = 171). However, this apparent difference was not significant. OMDS, Osame Motor Disability Score; P‐HAM, human T‐lymphotropic virus type 1‐associated myelopathy/tropical spastic paraparesis with parkinsonism; T‐HAM, typical human T‐lymphotropic virus type 1‐associated myelopathy/tropical spastic paraparesis (without parkinsonism).

We investigated whether there were clinical differences between P‐HAM and T‐HAM (Table 2). Of the 11 P‐HAM cases, 10 were females, and the onset age was significantly older compared with T‐HAM cases (65.0 vs. 48.8 years, *p* = 0.001, 95% confidence interval [CI] 6.0 to 25.0). Despite a shorter duration of illness (8.5 vs. 12.5 years, *p* = 0.151, 95% CI −8.0 to 1.0), the OMDS was significantly higher in P‐HAM cases than in T‐HAM cases (6.3 vs. 4.6, *p* = 0.0132, 95% CI 0.0 to 3.0), and all P‐HAM cases had OMDS ≥ 4. Motor symptoms were the most common initial presentation, with > 50% of patients in both groups first noticing gait disturbance. Two P‐HAM cases presented rapid progression; however, the frequency of patients with rapid disease progression did not significantly differ between the two patient groups. The time from illness onset to an OMDS of 4 also tended to be shorter in the P‐HAM group, although this apparent difference was not significant between the two groups (Figure 2). Approximately 50% of patients reached OMDS 4 in 7 years in the P‐HAM group and in 9 years in the T‐HAM group, according to the Kaplan–Meier analyses. Notably, despite the significantly higher OMDS in the P‐HAM cases, no differences in MRC sum scores were observed between the two groups when comparing lower limb strength alone or overall limb strength. These results indicate that P‐HAM cases have a higher age of onset than T‐HAM cases and comparable lower limb muscle strength. P‐HAM cases also appear to have greater disease severity relative to disease duration, which may suggest more rapid symptom progression.

**TABLE 2:** Clinical characteristics of P‐HAM and T‐HAM.

	P‐HAM, *N* = 11	T‐HAM, *N* = 235	*p*
Female ratio (%)	90.9% (1 males: 10 females)	73% (64 males, 171 females)	n.s.
Age at admission (years)	73.5 ± 5.8 (58–79)	61.3 ± 12.2 (15–87)	< 0.0001
Onset age of HAM/TSP (years)	65.0 ± 12.5 (40–77)	48.8 ± 17.3 (9–85)	0.001
Duration of HAM/TSP (years)	8.5 ± 10.3 (0–32)	12.5 ± 11.9 (0–59)	n.s.
Onset Age of Parkinsonism (years)	70.6 ± 5.3 (57–77)	N.A.	N.A.
Duration of Parkinsonism (years)	2.8 ± 2.8 (0–10)	N.A.	N.A.
Initial symptoms			
Gait (%)	54.50	52.30	n.s.
Urinary (%)	18.20	24.70	n.s.
Sensory (%)	0	11.10	n.s.
Others (%)	27	11.90	n.s.
Rapid disease progression (*n*)	2	30	n.s.
Osame Motor disability score	6.3 ± 2.5 (4–11)	4.6 ± 2.4 (0–13)	0.0132
Score more than 4 (*n*)	11 (100%)	173 (73.6%)	0.0489
MRC sum score			
Upper and lower limbs	54 ± 4.2 (49–59), *N* = 6	54.6 ± 6.3 (26–60), *N* = 109	0.3795
Lower limbs	25 ± 3.7 (19–29), *N* = 6	24.8 ± 6.0 (0–30), *N* = 109	0.6688

*Note:* Data are presented as the mean ± SD (range).

Abbreviations: MRC, Medical Research Council; N.A., not available; n.s., not significant; P‐HAM, human T‐lymphotropic virus type 1‐associated myelopathy/tropical spastic paraparesis with parkinsonism; T‐HAM, typical human T‐lymphotropic virus type 1‐associated myelopathy/tropical spastic paraparesis (without parkinsonism).

### 3.4 Comparison of Laboratory Findings Between P‐HAM and T‐HAM

To examine potential differences in viral infections and immune responses with the co‐occurrence of parkinsonism and HAM/TSP, we compared HTLV‐1 PVL and inflammatory markers between cases with P‐HAM and those with T‐HAM. The average HTLV‐1 PVL in peripheral blood mononuclear cells of P‐HAM cases was 7.96%, which did not significantly differ from that of T‐HAM cases (Table 3). An analysis of CSF markers also revealed no significant differences in cell number, protein levels, or neopterin concentrations between the two groups. Together, these results suggest that there are no differences in the degrees of viral infections and CNS inflammation between the two groups.

**TABLE 3:** Laboratory findings of P‐HAM and T‐HAM.

	P‐HAM, *N* = 11	T‐HAM, *N* = 235	*p*
HTLV‐1 PVL in PBMC (%)	7.96 ± 8.89, *N* = 7	9.03 ± 10.18, *N* = 104	n.s.
Cerebrospinal fluid			
Cell (/mm^3^)	2.2 ± 2.1	5.5 ± 9.3, *N* = 207	n.s.
Protein (mg/dL)	37.8 ± 17.1	39.6 ± 15.7, *N* = 224	n.s.
Neopterin (pmol/mL)	14.3 ± 11.9, *N* = 6	23 ± 37.7, *N* = 72	n.s.

*Note:* Data are presented as the mean ± SD.

Abbreviations: HTLV‐1, human T‐lymphotropic virus type 1; n.s., not significant; PBMC, peripheral blood mononuclear cell; P‐HAM, HTLV‐1‐associated myelopathy/tropical spastic paraparesis with parkinsonism; PVL, proviral load; T‐HAM, typical HTLV‐1‐associated myelopathy/tropical spastic paraparesis (without parkinsonism).

### 3.5 Treatment Responses in Patients With Parkinsonism and HAM/TSP

Patients with P‐HAM received treatment for both parkinsonism and HAM/TSP. As mentioned earlier in the Results, L‐DOPA was effective in nine out of 10 patients for motor symptoms including rigidity and bradykinesia. Furthermore, nine patients were treated with steroids for HAM/TSP symptoms, of whom eight demonstrated improved muscle strength or reduced gait disturbances, and one showed no improvement. The ineffective case may be attributed to a long disease duration and/or the irreversible nature of the neurological damage. Additionally, the two patients who were not treated with steroids because of mild symptoms or comorbidities were already receiving other oral immunotherapies.

## 4 Discussion

In the present study, 11 out of 246 HAM/TSP patients (4.5%) concurrently exhibited symptoms of parkinsonism and HAM/TSP. These P‐HAM patients presented various types of parkinsonism and had a later age of onset and greater disease severity than T‐HAM patients; however, they had no significant differences in lower limb muscle strength.

The severe motor impairment that occurs in P‐HAM is considered to result from the combined effects of parkinsonism‐related motor dysfunction and HAM/TSP symptoms. Patients with HAM/TSP generally present spasticity and hyperreflexia in the lower limbs. Because spasticity and hyperreflexia in parkinsonism typically follow an MSA‐like pattern [30], we anticipated that the combination of both would present MSA‐like clinical manifestations. However, the phenotypes of parkinsonism observed in our patients were diverse and did not show a consistent pattern. Regarding treatment, steroid therapy was administered to most patients and proved effective for improving lower muscle strength and gait disturbances. In addition, L‐DOPA therapy to treat parkinsonism led to improvements in motor impairments, including rigidity and bradykinesia. Their functional imaging showed dopaminergic neuron dysfunction. In HAM/TSP patients, considering the potential for parkinsonism as a complication, it is crucial to diagnose and treat both conditions accurately. In addition, although PD, MSA, PSP, and DLB were collectively analyzed as P‐HAM in this study, their clinical courses differ, and it will be important to investigate the distinctions among these types in future studies.

HTLV‐1 mainly infects CD4^+^ T cells, which infiltrate the CNS. Excessive immune responses, including HTLV‐1‐specific cytotoxic CD8^+^ T lymphocytes targeting the infiltrating infected cells, are considered to play a role in HAM/TSP pathogenesis, leading to bystander damage to neurons and glial cells [3]. Although the clinical manifestations of HAM/TSP suggest that the primary lesion is in the spinal cord, a neurohistological analysis revealed that chronic inflammation and diffuse degeneration are not confined to the spinal cord but also extend into the brain [31, 32, 33]. Furthermore, neuroimaging studies have demonstrated white matter lesions in MRI of patients with HAM/TSP, thus providing evidence of brain inflammation [17, 34]. In our study, there were no differences in PVL or neopterin levels between P‐HAM and T‐HAM, suggesting that CNS inflammation might persist in P‐HAM as well as T‐HAM. Viral infections may induce parkinsonism through mechanisms involving either direct or indirect damage to dopaminergic neurons. Influenza, herpes simplex virus 1, and Epstein–Barr virus are all viruses that are considered to cause parkinsonism via autoimmune mechanisms following infection [13, 14, 16]. Viruses can elicit autoimmune responses through various mechanisms, including molecular mimicry, bystander activation, and viral persistence, with or without epitope spreading. When virus‐specific T cells encounter infected cells, the infected cells present viral peptides via the major histocompatibility complex to CD8^+^ or CD4^+^ T cells. This triggers the release of cytokines, which may then cause the bystander killing of uninfected neighboring cells [35]. Recent studies have also reported an association between age‐related immune alterations, including immunosenescence and inflammaging caused by viral infections, and PD development [11]. In comorbid parkinsonism with HAM/TSP, the HTLV‐1 infection might drive an inflammatory response that affects dopaminergic neurons. Nonetheless, neuropathological studies are needed to determine whether chronic inflammation caused by HTLV‐1 infection contributes to the development of parkinsonism.

The present study revealed that 4.5% of HAM/TSP cases presented with parkinsonism, and 2% were complicated by PD. The prevalence of PD is generally 0.3%, although this rises to 1% in individuals over 70 years of age [8, 36]. The mean age of HAM/TSP patients in this study was 61.8 years, suggesting a relatively higher likelihood of parkinsonism complications in HAM/TSP patients compared with the general population. However, in the current study, we examined cases of HAM/TSP complicated by parkinsonism only. It is therefore essential to compare the incidence of PD in HTLV‐1‐infected carriers with that of the general population, to determine whether HTLV‐1 infection can contribute to parkinsonism.

**FIGURE 3:**
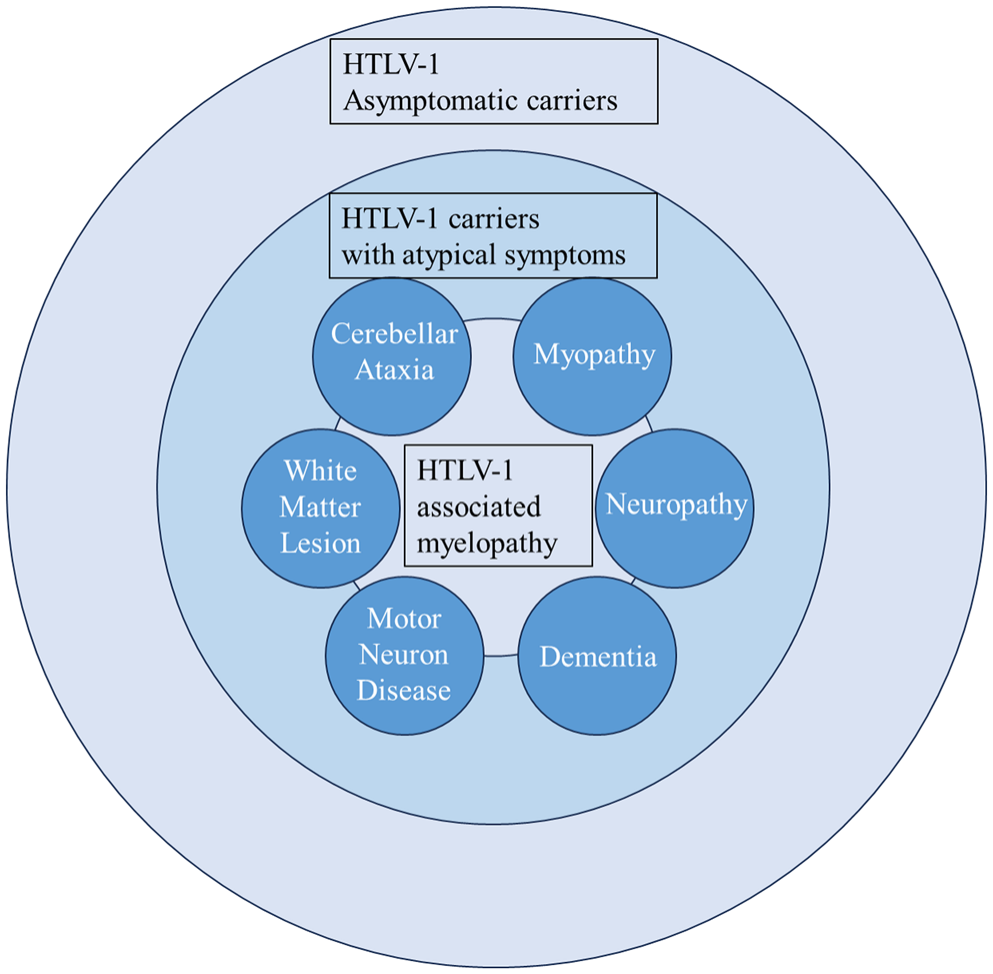
Spectrum of neurological disorders associated with HTLV‐1 infection. Relationship diagram of HAM patients, atypical symptoms, and asymptomatic carriers. HAM, HTLV‐1‐associated myelopathy; HTLV‐1, human T‐lymphotropic virus type 1.

In conclusion, our findings revealed that 4.5% of HAM/TSP patients exhibited a combination of parkinsonism and HAM/TSP‐related symptoms. These P‐HAM patients presented with various forms of parkinsonism. They also tended to have a later age of onset and greater severity over a shorter disease duration compared to T‐HAM cases, probably due to the additive effect of motor dysfunction related to parkinsonism on preexisting HAM/TSP symptoms. The coexistence of parkinsonism and HAM/TSP may be overlooked, and our findings help to define the clinical spectrum of neurological diseases associated with HTLV‐1 infection (Figure 3). Nonetheless, because the study was conducted at a single institution, our findings may reflect P‐HAM prevalence within a limited geographic region. Thus, a comprehensive global study on the incidence of parkinsonism in HTLV‐1 carriers will be valuable for understanding the spectrum of diseases associated with HTLV‐1 infection.

## Author Contributions

Mika Dozono and Satoshi Nozuma: drafting/revision of the manuscript for content, including medical writing for content; major role in the acquisition of data; study concept or design; analysis or interpretation of data. Shota Hirakata and Takashi Yoshida: major role in the acquisition of data; drafting/revision of the manuscript for content. Daisuke Kodama, Masakazu Tanaka, and Eiji Matsuura: drafting/revision of the manuscript for content. Ryuji Kubota and Hiroshi Takashima: drafting/revision of the manuscript for content, including medical writing for content; study concept or design; analysis or interpretation of data.

## Disclosure

The authors have nothing to report.

## Conflicts of Interest

The authors declare no conflicts of interest.

## Data Availability

The data that support the findings of this study are available on request from the corresponding author.
